# Cooperation of Genomic and Rapid Nongenomic Actions of Estrogens in Synaptic Plasticity

**DOI:** 10.1007/s12035-016-9979-y

**Published:** 2016-06-20

**Authors:** Yu-Jie Lai, Dan Yu, John H. Zhang, Guo-Jun Chen

**Affiliations:** 1 Department of Neurology, the First Affiliated Hospital of Chongqing Medical University, Chongqing Key Laboratory of Neurology, 1 Youyi Road, Chongqing, 400016 China; 2 Department of Neurology, Affiliated Haikou Hospital of Xiangya Medical College of Central South University, Haikou Municipal Hospital, Haikou, Hainan 570208 China; 3 Department of Anesthesiology, Loma Linda University School of Medicine, Loma Linda, CA 92354 USA

**Keywords:** Estrogens, Synaptic plasticity, Brain, Genomic, Nongenomic, Signaling cascades

## Abstract

Neuroplasticity refers to the changes in the molecular and cellular processes of neural circuits that occur in response to environmental experiences. Clinical and experimental studies have increasingly shown that estrogens participate in the neuroplasticity involved in cognition, behavior, and memory. It is generally accepted that estrogens exert their effects through genomic actions that occur over a period of hours to days. However, emerging evidence indicates that estrogens also rapidly influence the neural circuitry through nongenomic actions. In this review, we provide an overview of the genomic and nongenomic actions of estrogens and discuss how these actions may cooperate in synaptic plasticity. We then summarize the role of epigenetic modifications, synaptic protein synthesis, and posttranslational modifications, and the splice variants of estrogen receptors in the complicated network of estrogens. The combination of genomic and nongenomic mechanisms endows estrogens with considerable diversity in modulating neural functions including synaptic plasticity.

## Introduction

Neural circuit activity is essential for normal brain processes [1–3]. The fine regulation of this activity is driven by changes in synaptic structure and function, which is the cellular mechanism underlying cognition, behavior, and memory [4, 5]. Thereby, synaptic plasticity, which is the changes that occur in the number and/or morphology of neuronal synapses, is considered a fundamental feature of the nervous system, and it allows for adaptation to changing behavioral environments [6, 7]. Importantly, many of the cellular processes that contribute to synaptic plasticity are associated with neuronal disorders [8–10]. Understanding the abilities of synapses to modify their functional strength according to the environment and/or extracellular signals will help to delineate the underlying molecular mechanisms that allow these events to occur.

Estrogens are neuroactive steroids and/or neurosteroids that have the potential to influence the nervous system [11, 12]. Clinical and experimental studies have overwhelmingly demonstrated a modulatory role of estrogens in the brain and suggest their beneficial actions in neuronal plasticity [13–17]. For example, estrogen-primed animals exhibit decreased hippocampal seizure thresholds [18], and pretreatment with exogenous estrogens decrease rodent mortality and infarct size following middle cerebral artery occlusion [19–21]. Collectively, those studies reported an association between lower estrogen levels and increased risk of neuronal disorders which matched with human disease patterns that have long been known to differ between men and women and between premenopausal and postmenopausal women [22]. Consequently, interest in learning how estrogens affect the neuroplasticity of neural circuits and thus contribute to cognition, behavior, and memory is increasing. Thus, the underlying mechanisms of estrogenic action in neuroplasticity need to be clarified in order to better understand their functions in the normal activity of neural circuits and their potential therapeutic roles in brain disorders. In addition to the effects of estrogen on the regulation of gene transcription, which requires hours to days to manifest [5, 23], accumulating studies have shown that estrogens affect neuroplasticity within minutes [24–27]. Therefore, the relationships of estrogens with rapidly activated signaling cascades and transcriptional machinery and their potential crosstalk or convergence are evident [1, 28, 29].

Although genomic and nongenomic estrogenic effects are often viewed as distinct modes of estrogenic action, this may not necessarily hold true because the inhibition of one can limit the effectiveness of the other [30]. In this review, we focus on the relationship between the rapid nongenomic and genomic signaling of estrogens. First, we provide an overview of the advancements that have been made in the understanding of the rapid nongenomic and genomic signaling of estrogens in synaptic plasticity. Second, we discuss how the rapid nongenomic and genomic signaling actions of estrogens in the brain might rely on a cooperation or sequential modulatory system that ultimately results in changes in synaptic activity. Finally, we explore the molecular and cellular mechanisms that underlie the relationship between the rapid nongenomic and genomic signaling of estrogens.

## The Genomic Actions of Estrogens in Synaptic Plasticity

**Fig. 1:**
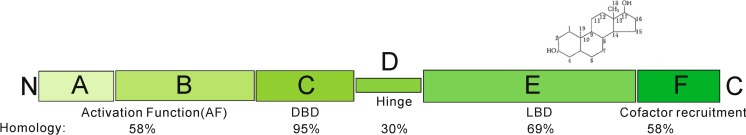
Primary structure of the classic estrogen receptors (ER). ERα and ERβ share a common structure with six functional domains (A/B, C, D, E, and F). Domain *A*/*B* plays a role in protein-protein interactions and the transcriptional activation of target gene expression. Domain *C* is responsible for DNA binding and ER dimerization. Domain *D*, which is a hinge domain linking domains *C* and *E*, is involved in the nuclear localization of ERs. Domain *E* is the ligand-binding domain. Domain *F* contains cofactor recruitment regions. The similar structures of the receptors both contain a highly homologous DNA-binding region (95 %) and a hormone-binding region with weaker homology(69 %). However, the carboxy- and amino-terminal regions have the least homology (58 %)

Estrogens have a variety of effects on the central nervous system (CNS). These effects, which typically require hours or even days to take effect, are mediated through transcriptionally regulated changes in gene expression [31]. These estrogenic actions, which have been described as genomic effects, are characterized by the following features: a prolonged latency; long lasting; involving gene transcription and protein synthesis; initiation within the nucleus, usually through the nuclear receptor superfamily; ineffectiveness of steroid analogs that are unable to cross the plasma membrane [32, 33]; and functional at physiological levels [34]. Nuclear estrogen receptors (ERs) have two forms: ERα and ERβ [35–37]. Like other members of the nuclear receptor superfamily, ERα and ERβ contain a common structure of six functional domains (A/B, C, D, E, and F), as shown in Fig. 1 [36, 38]. In the inactive state, ERs usually exist as a monomer or complex with immunophilins and heat shock protein 90 (HSP-90) [39]. The estrogen-ER complex then binds to the estrogen response element (ERE) in the promoter region of the target genes and exerts its regulatory potential [40, 41].

Based upon the findings of previous studies on the genomic effects of estrogens on synaptic plasticity [39], novel research techniques, including pharmacological and gene manipulation, have recently been applied to better understand these genomic effects. *HOXC10*, which is one of the few neural gene targets of ERs, has been shown to play a critical role in spinal cord development and neuron formation [42, 43]. The authors of those studies found that the *HOXC10* promoter contains multiple putative EREs, which indicates that *HOXC10* might be transcriptionally regulated by estrogens. In addition, they showed that the ERE1 and ERE6 regions of the *HOXC10* promoter are potentially involved in the transcriptional regulation of *HOXC10* expression in the presence of estrogens [44]. Another neural gene target of ERs is *Apo D*, which encodes apolipoprotein D. Many studies have demonstrated a relationship between neuronal degeneration and *Apo D* expression [45, 46]. A recent study demonstrated that *Apo D* has three EREs in its promoter and that its expression can be modulated by these hormones [47]. Thus, these findings suggest that *Apo D* is partly responsible for the neuroprotective role of estrogens. In addition, the large-conductance voltage- and Ca(2+)-activated K(+) channel has been shown to play key roles in diverse body functions that are influenced by estrogens. The pore-forming alpha subunit (*Slo, KCNMA1*) promoters of the K(+) channel contain multiple EREs. A mutagenesis experiment further showed that the estrogen responsiveness of the *mSlo* gene involves a classical genomic mechanism that acts through ERE1 and ERE2 and that is facilitated by ERα, which therefore suggests that ERα exerts a genomic action on the *mSlo* gene promoter elements [48]. Luckily, the identification of estrogen target genes is greatly facilitated by transcriptomic methods, such as RNA sequencing, expression microarrays, and chromatin immunoprecipitation (ChIP) with massively parallel DNA sequencing (ChIP sequencing). Combining transcriptomic and ChIP sequencing data enables the discrimination of direct and indirect estrogen target genes [49]. Interestingly, Humphreys et al. analyzed the transcription of control and estradiol (E2)-treated animals with RNA sequencing and found significant alterations in the transcript levels of 88 genes in the treated animals [50]. Another gene assay and cell-based endogenous expression analysis revealed that estrogens significantly suppress the expression of brain size-related genes, including *MCPH1*, *ASPM*, *CDK, RAP2*, and *WDR62* [51]. Intriguingly, when the EREs are deleted from the promoters, the suppressive effects are abolished, which suggests that EREs mediate the effects of estrogens on the brain size genes [51]. Ryokoet al. identified a classical ERE half-site on a *TPH2* gene promoter that is functional because the deletion or mutation of this sequence blocks the E2-induced *TPH2*-luc activity. These results suggest that the ERE half-site plays an important role in the ER-mediated regulation of *TPH2* transcriptional activity [52]. Hence, these studies indicate that advances in genomic technology allow for the identification of the neuronal target genes of estrogens and ERE-binding sites.

**Fig. 2:**
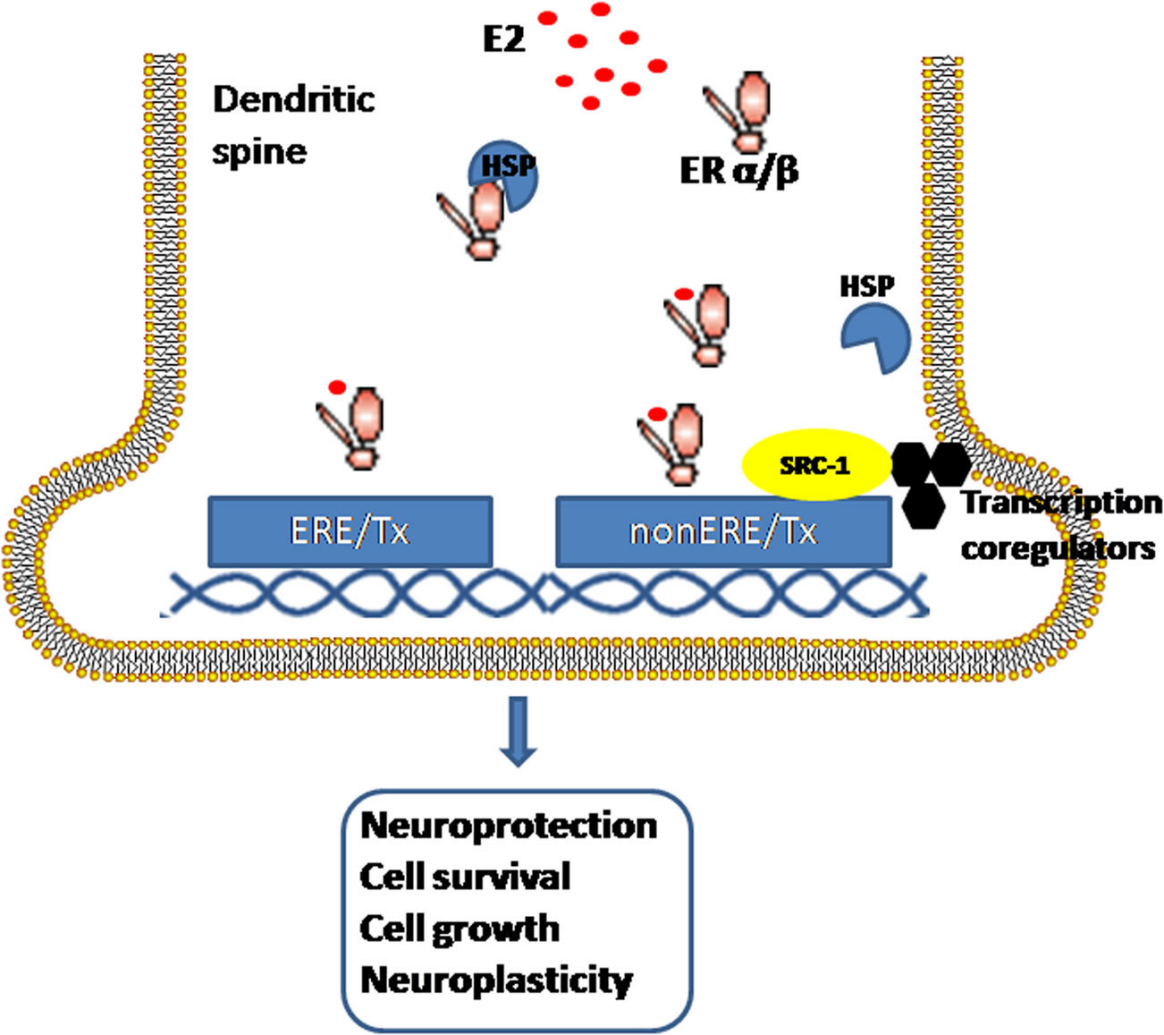
Schematic of the genomic estrogenic actions in neurons. The estrogen-ER complex binds to the estrogen response element (ERE) in the promoter region of target genes or acts as a cofactor/coregulator at non-ERE sites that interact with other DNA-binding elements. *E2*, estradiol; *SRC-1*, steroid receptor coactivator-1

In addition to the mechanisms described above, estrogens serve as cofactors at non-ERE sites that interact with other DNA-binding elements, such as AP-1 or c-Jun [38]. However, ERs modulate chromatin states by associating with different classes of coregulators [53]. Steroid receptor coactivator-1 (SRC-1) is the predominant coactivator of p160 family members in the brain. Several studies have shown that the expression of SRC-1 in the hippocampus is highly correlated with several key synaptic proteins during development or after orchidectomies, but not after ovariectomies. These findings indicate that SRC-1 may be regulated by hippocampal-synthesized E2 and profoundly involved in the hippocampal E2 regulation of hippocampal synaptic plasticity [54]. While the roles of a large number of activator complexes and their associated enzymatic activities have been well established by the presence of the histone acetyltransferases (HATs) p300/CBP and the levels of H3K4me2 and H3K27Ac [55], the precise biochemical mechanisms by which so many of the coactivators that are required for the different functional activities are recruited at specific enhancer sites remain incompletely understood [56, 57]. Liu et al. reported a new signature of the functionally active estrogen-regulated enhancers that involve the selective trans-recruitment of an apparent complex of other DNA-binding transcription factors, including RARα/γ, GATA3, AP2γ, STAT1, AP1, and FoxA1 [58]. However, this phenomenon has only been described in MCF7 cells. Whether a similar conclusion can be made about the brain is still unclear (Fig. 2).

Notably, both ERα and ERβ are abundantly localized throughout the brain, but the relative local distributions of ERα and ERβ may differ [35, 59]. The differential local distribution of ERs most likely indicates the different effects of local E2 administration in the brain [60]. Evidence has revealed that the individual or simultaneous activation of ERα and ERβ has different effects [61]. These findings suggest the intriguing hypothesis that the interactions between the two ERs and the consequences of transcriptional modulation are complicated and delicately balanced. Several studies have reported a phenomenon of an opposite transcriptional response between ERα and ERβ that depended on the cellular context and associated cofactors [62, 63]. However, the exact relationship of ERα and ERβ and the precise actions of their interaction remain largely unknown due to a lack of systemic investigations and related methods.

## The Rapid Nongenomic Actions of Estrogens in Synaptic Plasticity and Neuroprotection

Evidence that has been collected during the last decade has indicated that estrogens elicit cellular actions that occur as fast as seconds to minutes and mostly within 1 h [64, 65]. In vitro studies have shown that estrogens acutely modulate synaptic function in both sexes. Methods that are more sensitive have detected the subcellular location of ERs [66, 67], including nuclei, cytoplasm, plasma membranes, perimembrane spaces, endoplasmic reticuli, and mitochondria. Notably, the ERs located outside of the nucleus are suspected to be related to the rapid actions of estrogens [22]. Except for the established ERα and/or ERβ, there are at least three putative ERs that are involved in the rapid actions of estrogens in the brain: G protein-coupled receptor 30, G protein-coupled estrogen receptor 1, and the membrane-associated ER (mER) [68–70]. The putative ERs have the characteristics of initiating cell signaling from the membrane [71] with a companion G protein-coupled receptor [72]. Moreover, a high-affinity, saturable, and 3H-estradiol-binding site in the plasma membrane has been identified and designated as ER-X [69]. Using a novel putative mER agonist STX, it was reported that rapid membrane ER activation can initiate cellular signaling through the metabotropic glutamate receptor (mGluR) 1a [71]. Study also shows that the mER (not ERα or ERβ) mediates the estrogen-initiated inhibition of the expression of the ubiquitin-conjugating enzyme 9, which is the only known E2-conjugating enzyme and which is associated with neuroplasticity [73]. Therefore, the extranuclear receptors are thought to be essential for mediating the rapid nongenomic actions of estrogens. However, these putative ERs have not been structurally characterized [69].

A major debate on whether the circulating estrogens and/or local estrogens that are derived from the brain underlie the rapid effects of estrogens has existed for many years [74, 75]. Recent reports have demonstrated that the concentrations of estrogens are at nanomolar levels in some brain regions and at picomolar levels in the plasma [76]. Thus, the concentrations of circulating estrogens may be too low to initiate rapid actions [77]. These observations have suggested that the levels of the local estrogens that are synthesized within the brain, which fluctuate more rapidly than the levels of the circulating estrogens, play an essential role in the rapid actions of estrogens. This claim has been strengthened by the discovery of multiple enzymes that allow for the biosynthesis of brain-derived estrogens [78–81]. Estrogens undergo tissue and/or cell-specific enzymatic conversions into estrogen metabolites [82]. The neurons that express many estrogenic enzymes are considered important producers of brain estrogens.

Several key enzymes that are involved in the synthesis of testosterone and estrogens in the brain use dehydroepiandrosterone as a precursor [83]. For example, the rate-limiting step of steroidogenesis is mediated by the steroid acute regulatory protein and transporter protein, which are widely found in typical steroidogenic tissue (e.g., ovary and adrenal gland) as well as in neurons [84]. Increasingly, a number of other enzymes, including the estrogen-synthesizing enzyme, aromatase, have been widely found in different brain regions of male and female rats [85]. 17β-Hydroxysteroid dehydrogenase type 10 (17β-HSD10), which is encoded by the *HSD17B10* gene that maps to *Xp11.2*, is a homotetrameric mitochondrial multifunctional enzyme that catalyzes the oxidation of neuroactive steroids. The brains of individuals with Alzheimer’s disease (AD) and animals in an AD mouse model exhibit abnormally increased levels of 17β-HSD10. The restoration of steroid homeostasis could be achieved through the supplementation of neuroactive steroids with a properly dosed treatment regimen or through the adjustment of 17β-HSD10 activity to protect neurons [86]. In addition, steroid sulfatase plays a key role in the intracrine conversion of dehydroepiandrosterone testosterone and estrogens [83]. Therefore, because neurons are equipped with all of the enzymes that are activated in steroidogenesis, they are therefore capable of synthesizing the so-called neurosteroids, which act directly and locally on neurons to exert neuroprotection. It is important to note that many of the enzymes involved in steroidogenesis are expressed in not only neurons in different brain regions but also astrocytes and even endothelial cells and oligodendrocytes [84]. It will be important to examine the astrocyte- and/or endothelial-specific enzymes in order to better clarify the contribution and/or crosstalk of these enzymes in non-neural cell types to the rapid actions of estrogens. Interestingly, a growing number of studies have found that the enzymatic activity that is responsible for the synthesis of estrogens can be modulated within minutes by mechanisms that cannot possibly involve changes in the concentrations of the enzymatic proteins [87, 88]. This mechanism underlying the rapid synthesis and metabolism of estrogens corresponds with the rapid action of estrogens in the brain. Collectively, these findings show that the presence and regulation of estrogenic enzymes in the brain and the extracellular localization of ERs are evidence of the rapid nongenomic actions of estrogens in synaptic plasticity.

To clarify the mechanisms through which estrogens exert their rapid effects on neuronal plasticity, two concepts need to be emphasized. The rapid nongenomic pathway is mediated by nonnuclear ERs and/or nuclear ERs [54]. The rapid signaling pathway whereby local estrogens modulate synaptic plasticity activate several cellular kinases, often through ion channels [89]. We use the term rapid nongenomic pathway to refer to processes that are coupled to the signaling cascade that requires ER participation, while rapid signaling pathway refers to the processes that are coupled to the signaling cascade in an ER-independent manner.

### The Actions of the Rapid Nongenomic Pathway of Estrogens in Synaptic Plasticity

The rapid nongenomic pathway of estrogens in the nervous system usually involves the activation of multiple kinase pathways, including the mitogen-activated protein kinase (MAPK)/extracellular regulated kinase (ERK) pathway, phospholipase C pathway, phosphatidylinositol 3-kinase(PI3K)/Akt pathway, and protein kinase A (PKA) and protein kinase C (PKC) pathways [5, 21, 90]. Estrogens rapidly enhance ERK and Akt activation through phosphorylation in cortical neurons, and inhibitors of ERK and Akt activation significantly attenuate estrogen induction in excitatory glutamatergic synapses [91, 92]. Once Akt and ERK are activated, many essential cellular functions, such as survival, adhesion, metabolism, and proliferation, are initiated [93]. Interestingly, the ability of estrogens to phosphorylate ERK and Akt persists, even in ERα-knockout mice, thus implicating other ERs in these estrogen actions [94]. Another potential example of an estrogen-induced rapid nongenomic pathway involves the regulation of the JNK-c-jun signaling pathway which was well recognized as pro-apoptotic factors in ischemic brain [95–97]. The rapid nongenomic pathway of estrogens was also mimicked by the application of the PKA activator forskolin and the PKC activator phorbol-12,13-dibutyrate. In addition, the rapid nongenomic pathway mechanisms of estrogens include the interactions of estrogens with the signaling of neurotrophic factors, such as brain-derived neurotrophic factors, insulin-like growth factor-1 (IGF), and Wnt. A novel model of the interactions of estrogens and acute brain-derived neurotrophic factor signals suggests that they act in a cooperative manner, which results in dendritic spine formation and the subsequent stabilization of synaptic and circuit plasticity [98, 99]. Moreover, ERα regulates the IGF-type I receptor (IGF-IR) signaling pathways through the phosphorylation of ERK and Akt, and the interactions of the ER-IGF-IR pathway potentiates neural activities [100]. Taken together, these findings indicate that the interactions of ERs and IGF-IRs are one of the important mechanisms underlying the rapid nongenomic pathways of ERs [100]. Therefore, estrogens can be coupled with neurotrophin receptors, which results in the convergence or cross-coupling of specific signaling pathways, particularly at the level of the MAPK cascade [94].

Along these lines, the rapid nongenomic pathway of estrogens can be presumed to be involved in the interactions of membrane-localized ERs with adaptor proteins, such as c-Src, and the downstream rapid signaling that occurs through the MAPK pathway, G proteins, PKA/PI3K pathway, or PKC pathway. These signaling pathways are rapidly activated, and they in turn trigger intracellular Ca^2+^ release, cAMP production, and/or c-Src activation with the subsequent activation of MAPK or calcium/calmodulin-dependent kinases. However, it is important to note that different receptors may be coupled with specific rapid signaling pathways in order to exert relative effects. The use of selective ER modulators and transgenesis (knockout and/or knockdown) mice in studies will help to clarify these issues.

Another question that should be addressed is how ERs couple with downstream cascade signaling. Intriguingly, outside the nervous system, ERα has been demonstrated to physically interact with CAV1, which is necessary for the trafficking of ERα to the membrane surface [101]. A subsequent study showed that membrane-localized ERs are localized within distinct caveolae and that CAV1 is necessary to couple ERα to the group I mGluRs and CAV3 is necessary for the association of ERα and ERβ with group II mGluRs [102]. However, recent studies on ER-interacting scaffold proteins have demonstrated that scaffold proteins, such as MNAR/PELP1, striatin, and p130Cas, might link ERs with kinases to potentially mediate estrogen-induced kinase signaling [103, 104].

### The Actions of the Rapid Signaling Pathway of Estrogens in Synaptic Plasticity

**Fig. 3:**
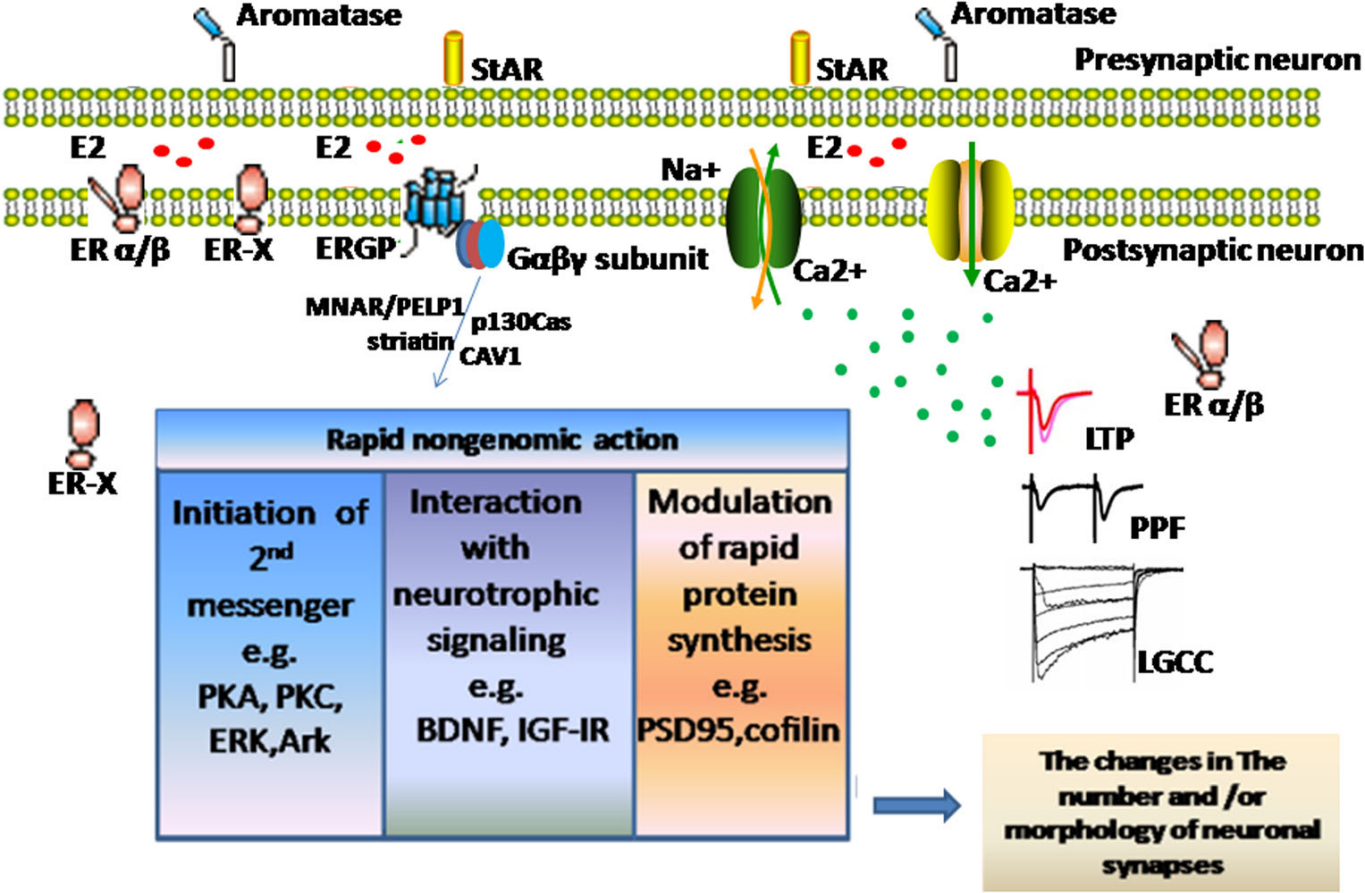
Schematic of the rapid nongenomic estrogenic actions in neurons. The expanded ERs are expressed throughout neurons, including the nuclei, cytoplasm, plasma membranes, perimembrane spaces, endoplasmic reticulum, and mitochondria. Local synthesis of estrogens, which is mediated by synaptic aromatase/steroid acute regulatory protein (StAR) or peripheral estrogens, results in the activation of ERs, ion channels, and/or other membrane receptors. Such activation couples with specific signaling cascades or second messenger systems through scaffold proteins (CAV/MNAR/PELP1/striatin/p130Cas) and ultimately leads to the remodeling of synaptic structure and function. *ERGP*, estrogen receptor G-protein; *LTP*, long-time potential; *LGCC*, L-type gate calcium channel

Estrogens rapidly potentiate kinate-induced currents in hippocampal neurons from wild-type as well as ER knockout mice, thus suggesting that estrogens directly interact with ion channels in synapses then regulate the downstream signaling cascades [105–107]. L-type voltage-gated Ca^2+^channels (VGCCs) play important roles in dendritic development, neuronal survival, and synaptic plasticity [108]. In electrophysiological studies, estrogens acutely potentiate VGCCs in hippocampal neurons in an ER-independent manner by directly binding with a domain that overlaps with the dihydropyridine-binding site [17]. Calcium/calmodulin-dependent protein kinase-II (CaMKII) is a major neuronal protein that plays a significant role in the cellular processes of long-term potentiation (LTP) and the vesicular release of neurotransmitters [109, 110]. The loss of CaMKII in the forebrain has severe adverse effects on spatial learning in mice [110]. The activation of CaMKII by estrogen-modulated LTP induction stimulates the formation of new spines and enlarges existing spines [111, 112]. However, this effect of estrogens is not mediated by ER-dependent actions [113]. A number of reports have indicated that proline-rich tyrosine kinase 2(PYK2), which is a redox-sensitive kinase, is activated by estrogen [114]. The activation of PYK2 in cerebral ischemia is involved in the modulation of *N*-methyl-d-aspartate-type glutamate receptor activity and Ca^2+^ dynamics, which result in ischemic neuron death. However, the detailed mechanisms underlying the PYK2 activation by estrogens remain unclear. Several studies have shown that estrogens are able to interact with K^+^ channels in different types of cells, such as cardiac myocytes and neurons [115, 116]. Furthermore, tyrosine kinases seem to be involved in the activation of volume-sensitive K(+) channels, whereas tyrosine phosphatases appear to be involved in the inactivation of channels by estrogens [116–118]. Collectively, these findings suggest that estrogen regulation of PYK2 is mediated by direct interaction with potassium channels. Until recently, no evidence existed that estrogens modulate mGluRs directly without ERs [101, 119, 120]. Through these rapid pathways, estrogens play an important role in the modulation of synaptic plasticity (Fig. 3).

## Cooperation of the Genomic and Rapid Nongenomic Actions of Estrogens in Synaptic Plasticity

**Fig. 4:**
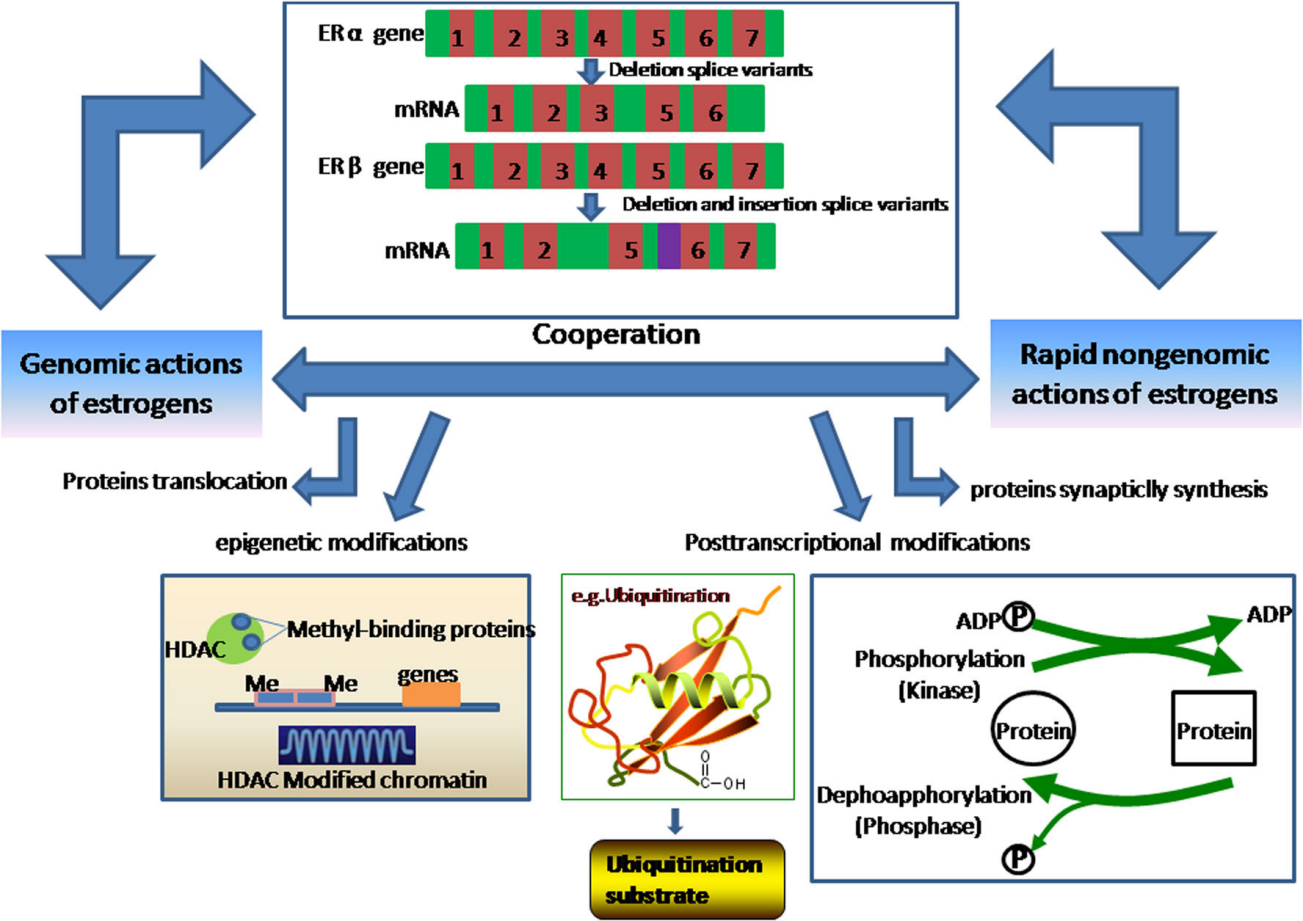
Schematic of the cooperation of the genomic and rapid nongenomic actions of estrogens in neurons. The cooperation of the genomic and rapid nongenomic actions of estrogens is involved in synaptic plasticity. Epigenetic modifications, synaptic protein synthesis, posttranslational modifications, and ER splice variants are thought to be the main molecular mechanisms that underlie the cooperation of the genomic and rapid nongenomic actions of estrogens

In the classical mechanism, estrogens bind to the α or β isoform of the ERs, which then binds to EREs and alters gene transcription [31]. The nonclassical actions of ERα and ERβ possibly occur through alternative response elements in DNA or rapid changes in signaling cascades [121, 122]. All of these mechanisms operate in the CNS [123]. The nuclear accumulation of ERβ, which occurs 6–12 h after estrogen treatment, results in the increased expression of postsynaptic density (PSD)-95 and synaptophysin messenger RNA (mRNA), thus implicating the classical genomic estrogenic actions on synaptic plasticity. However, blocking PI3K signaling partially suppresses the estrogen-induced expression of PSD-95 and synaptophysin, which suggests a crosstalk between the genomic and nongenomic actions of estrogens [124]. As shown in Fig. 4, specific cellular and molecular mechanisms underlie the cooperation of genomic and rapid nongenomic actions of estrogens [125]. Previous studies have indicated that alternative rapid signaling cascades of estrogens act by interfering with activation of the ERK and PI3K signaling pathways that regulate on a transcriptional level [126, 127]. One of the most intensely studied mechanisms involves antiapoptotic genes, such as *BCL-2*. Several studies have shown that estrogens activate different pathways to modulate *BCL-2* transcription. Wu et al. showed that estrogen induces rapid Ca2+ influx in hippocampal neurons, which results in the activation of the Scr/ERK signaling cascades and the upregulation of *BCL-2* transcription [128]. Pugazhenthi et al. reported that estrogens regulate the activation of Akt/PKB pathways, which induce the expression of *BCL-2* mRNA through the phosphorylation of the cAMP response element (CRE) binding (CREB) protein and its subsequent binding to the CRE in the *BCL-2* promoter [129]. Indeed, estrogens are involved in the regulation of pCREB within 15 min of estrogen application, thus indicating the rapid involvement of multiple signaling pathways, such as Ca^2+^, CaMKII, PKA, and/or ERK [130]. Hence, we speculate that CREB is a critical transcription factor in the regulation of the transcription of many genes and a target for nonclassical estrogen signaling, which provides the most direct relationship between rapidly activated signaling cascades and transcriptional mechanisms [131]. Such systems underlie many regulatory mechanisms of synaptic plasticity in the brain, particularly the regulation of synaptic protein expression [132]. Estrogens regulate biphasic *NPY* gene expression at the level of the NPY promoter. However, the rapid nongenomic actions of estrogens, which are linked to the PI3K/Akt and ERK/MAPK pathways, are critical in this process. The corresponding pharmacological inhibitors of the PI3K/Akt or ERK/MAPK pathways block the effect of estrogens on *NPY* gene expression. These observations suggest that the rapid signaling events that are induced by estrogens potentiate the genomic actions of estrogens on *NPY* gene expression [30]. Although not all estrogenic mechanisms for modulating gene transcription depend on the rapid nongenomic effects and signaling pathways, a solid and classical mechanism underlies the cooperation of the rapid nongenomic and genomic signaling of estrogens in neuronal plasticity and neuroprotection. Notably, three sequences that appear to be EREs have been found in the promoter region of rat Nav1.7 (SCN9A). Similarly, two ERE-like sequences have been found in the promoter region of human Nav1.7 (SCN9A). Therefore, it is highly likely that estrogens regulate Nav1.7 mRNA expression [133]. It is also important to note that activation of estrogen receptor rapidly rescues the impairment of neuronal excitability through BK K+ channel-mediated mechanism in brain slices after oxygen-glucose deprivation [134], suggesting that this rapid signaling is also neuroprotective. Consistently, the increased BK K+ channel-mediated currents and related mRNA levels are found in neuronal cells treated with physiological concentrations of E2 [135]. Because of the important role of ion channels in the activation of signaling cascades, we hypothesize that the genomic effects of estrogens may also remodulate their rapid signaling cascades.

### The Role of Epigenetic Modifications

The alterations in neuronal gene expression that result from the classical genomic actions of estrogens play an important role in neuroplasticity and neuroprotection, as described above. However, the current understanding of pretranscriptional regulatory processes is poor [136]. A growing number of lines of evidence indicate that epigenetic mechanisms regulate transcription without modifying gene sequences in memory formation, neuroplasticity, and neurodegeneration [137]. Indeed, extracellular cues, including synaptic activity, and neurotrophic factors might crosstalk with the neuronal transcriptional response through epigenetic modifications [138]. Histone phosphorylation or acetylation and DNA methylation are the main epigenetic mechanisms that control gene expression through modification of the chromatin structure, and these mechanisms are critically involved in the regulation of nuclear receptor-mediated transcription [139]. Indeed, the rapid signals that are initiated by estrogens impact the epigenetic modifications that are thought to be involved in the consolidation of memory and neuroplasticity [136, 140, 141].

It was reported that responses to estrogens involved highly specific changes in epigenetic modifications, dependent on cell group, gene, histone modification studied, promoter/enhancer site, and time following estrogen treatment [142]. A subsequent investigation showed that the memory-enhancing effects of estrogens are blocked by a potent HAT (whole name) inhibitor in vitro. In addition, the HAT inhibitor reverses the estrogen-induced increases in histone H3 acetylation, HAT activity, and levels of the de novo methyltransferase DNMT3B as well as the estrogen-induced decrease in the levels of the memory repressor protein histone deacetylase 2 [143]. Estrogens increase the histone H3 acetylation of target genes in the brain through its rapid signaling effects on the activation of ERK, which is essential for the consolidation of memory and neuroplasticity [144]. Wong et al. revealed that estrogens induce nongenomic ER signaling to activate PI3K/AKT, which results in AKT phosphorylation and inactivation of the histone methyltransferase EZH2, thus providing a direct link to disruption of the epigenome [145]. Although a variety of rapid nongenomic ER signals are involved in the epigenetic modifications of histone, estrogens significantly decrease the levels of expression of histone deacetylase. However, it is not clear if the mechanism is related to the rapid signaling actions of estrogens [146]. DNA methylation at CpG dinucleotides and two other alternative forms of methylation, non-CpG methylation and hydroxymethylation, has been reported in neurons [147, 148]. Altered DNA methylation in the brain has been implicated in epilepsy and AD [149, 150]. Thirty minutes after a single infusion of estrogens in the hippocampus, the infusion induced DNA methylation-dependent alterations in the transcription of immediate early genes and initiated a cascade of transcription factors, which contributed to long-term neuronal and circuit alterations [146]. Interestingly, the time course of the estrogen alterations of DNA methylation overlap with its rapid cellular actions, which occur as fast as seconds to minutes but mostly within 1 h. Therefore, it is likely that the activation of rapid signaling by estrogens promotes epigenetic reprogramming. Bredfeldtet al. demonstrated that the ER-mediated signaling that occurs through the PI3K/Akt pathway results in the phosphorylation of EZH2, which reduces the levels of methyltransferases [151, 152]. However, it is not clear if such mechanisms exist in the brain. Nonetheless, the results of these studies provide compelling evidence that communication between extracellular stimuli and chromatin occurs through the signal transduction pathways of estrogens. The mechanism for the rapid suppression of gene expression occurs through the epigenetic modification of methylation at the promoter regions [153]. In neurons, ERα expression rapidly increases after middle cerebral artery occlusion, which suggests a return to the developmental program of gene expression. The ERα gene is also methylated after neuronal injury, which suggests a role of DNA methylation in the regulation of ER expression in the brain. In addition, estrogens can induce differences in the DNA methylation of ERs in the brain [154]. Consequently, the altered expression of ERs in neurons may result in changes in its downstream effectors in both genomic and nongenomic pathways. However, these types of studies are still in their early stages, and advances are needed in the understanding of the hormonal regulation of the enzymes that control acetylation and methylation, transient versus stable DNA methylation patterns, and sex differences across the epigenome in order to fully understand the involvement of epigenetic modifications in brain function and behavior.

### The Role of Synaptic Protein Synthesis and Posttranslational Modifications

Synapses are dynamic structures that are continually shaped and remodeled by a rich variety of highly sophisticated protein complexes called scaffold proteins [155]. Many investigations have shown that estrogens affect synaptic scaffold proteins [156, 157]. Increases in spine density involve the formation of new spines, which thus requires the synthesis of new proteins within the PSD [158, 159].The consolidation of long-term synaptic plasticity requires de novo protein synthesis, which involves a mechanism that modulates the translation of a subset of neuronal mRNAs [160, 161]. Estrogens induce the rapid protein synthesis of new PSD-95, but they do not cause a rapid and significant increase in PSD-95 mRNA levels [162]. This observation suggests that estrogens may modulate the de novo synthesis of a variety of scaffold proteins in synapses in accordance with neuronal plasticity. One of the signature actions of estrogens is to alter the morphology of neural processes [163]. Dendritic remodeling requires structural modifications of the cytoskeletal protein β-actin. The severing of filamentous actin is performed by the constitutively active enzyme cofilin, which is inactivated by phosphorylation. Membrane-initiated estrogen signaling that involves the mGluR 1 is responsible for the phosphorylation and subsequent deactivation of cofilin [164]. Thus, this phenomenon suggests that estrogens enhance the PTMs of cofilin. PTMs are ubiquitously involved in complex neuronal processing and are well-established general mechanisms required for learning and memory as well as the underlying cellular correlate, long-term synaptic plasticity [165]. Another study reported that rapid and transient ER signaling stimulation affected the PTMs of RGSz1 protein isoforms, which thereby attenuated 5-HT_1A_R signaling in the hypothalamus [166]. A wide variety of neurodegenerative diseases that involve impairments in the ubiquitin-proteasome system have been described as proteinopathies that are caused by aggregate-prone proteins that are not efficiently removed by proteasomes [167]. The treatment of cells with estrogens results in aggregate removal and increased cell survival due to activation of the autophagic pathway. Interestingly, previous observations have suggested that estrogens enhance the ubiquitination of calcium channels, which decreases Ca^2+^ influx. Such actions reversely activate different signaling cascades that mediate rapid estrogenic actions. Consequently, it is reasonable to presume that the regulation of PTM mechanisms by estrogens links the cooperation of the genomic and rapid nongenomic actions of estrogens in synaptic plasticity and neuroprotection.

Interestingly, the findings of recent studies have suggested a novel model whereby the concomitant translocation of proteins from the dendritic cytoplasm to synapses and the nucleus occurs in response to synaptic activity in order to control neuronal plasticity [168]. However, whether estrogens acutely regulate such a model at adult synapses and if such effects are responsible for its actions that link its genomic and rapid nongenomic effects are still unknown. Several synaptically synthesized proteins that are transported to the nucleus from synapses may link specific types of stimuli with the nucleus, which indicates that the synapse-to-nuclear transport of proteins dynamically informs the nucleus about synaptic activity [169, 170]. Signals generated at synapses trigger transcriptional changes that is essential for neuronal development, required for persistent forms of learning-related synaptic plasticity [171, 172]. However, the precise mechanisms underlying the information transfer from the cytoplasm to the nucleus of neurons are still poorly characterized [173]. Estrogens increase the concentration of filamentous actin in spines and strongly enhance its polymerization in association with LTP. A study of the origins of these effects showed that estrogens activate the small GTPase RhoA and phosphorylate (inactivate) the actin-severing protein cofilin, which is a downstream target of RhoA. Moreover, an antagonist of RhoA kinase (ROCK) blocks estrogens’ synaptic effects. Estrogens thus emerge as a positive modulator of the RhoA-cofilin pathway that regulates the subsynaptic cytoskeleton. Moreover, ovariectomies decrease RhoA activity, spine cytoskeletal plasticity, and LTP, whereas brief infusions of estrogens rescue plasticity, thus suggesting that the deficits in plasticity arise from acute as well as genomic consequences of hormone loss [174]. Therefore, estrogens affect synaptic physiology by partially activating the actin-signaling pathways that may participate in the translocation of synaptic proteins [175]. Such a mechanism acts in the ERs themselves, as in estrogen-treated cells, and the membrane and cytosolic ERβ levels gradually decrease, while those of nuclear ERβ progressively increase in a time-dependent manner, thus suggesting the estrogen-dependent nuclear translocation of ERβ [124]. Although the data strongly support a role of this pathway in the control of the translocation of proteins from the dendritic cytoplasm to synapses and the nucleus, the direct regulation has not been tested.

### The Role of ER Splice Variants

The identification of multiple splice variants of ERs in rodents and humans has added a further layer of complexity to genomic regulation by estrogens [176]. In the hypothalamus, splice variants have been suggested to participate in membrane-initiated estrogenic signaling, which would connect the genomic and rapid nongenomic estrogenic effects [177]. The brain has the highest levels of exon skipping, which is the most common mechanism of alternative splicing [178, 179]. Canonical ER mRNA exons may generate a number of splice variants with single or multiple exon skipping, exon duplication, inserts, or partial exon deletions [180]. To date, the functional implications of ER splice variants in the CNS remain to be determined [3]. ERα splice variants have been investigated in the brain areas that regulate memory formation and that are affected in patients with AD [180]. The major ERα splice variants are the dominant negative del.7 isoform lacking exon 7, which encodes a substantial portion of the ERα ligand-binding domain. This alternative may protect tissues from excessive estrogenic effects [181]. However, the high concentrations of the dominant negative isoform del.7 that inhibits estrogenic signaling in the brains of the elderly and patients with AD suggest a possible reduction of the effects of estrogens on cognitive functions [180]. Importantly, the expression of multiple ER variants might be tissue-specific [182]. Indeed, Ishunina et al. found that the major ERα splice variant in the hippocampus was del.4 [183]. It is also worth noting that the ER splice variants were substantively different in structure. These structural differences suggest the intriguing possibility that these receptors may have specific functions not dependent on ligands and/or resistant to normal estrogenic effects, which could have detrimental consequences on therapeutic hormone treatment strategies during menopause or in disease states [184]. In addition to ERα splice variants, recent studies have identified those of ERβ in the CNS [60].

To date, three major splice variants of the classic ERβ receptor have been described in the rodent. These include a deletion of exon3, a deletion of exon 4, and an insert between exons 5 and 6 [185]. The human ERβ variants that have been identified to date contain variable length deletions and substitutions in exon 8 [176]. A novel finding demonstrated that human-specific ERβ splice variants exhibit marked constitutive activity in neuronal cells at both minimal and complex promoters. Furthermore, human-specific ERβ splice variants are largely unresponsive to ligands and induce modest increases of ERE-mediated promoter activity and robust decreases in AP-1-mediated promoter activity. Although the changes in the ERE-mediated promoter activity were modest, these fine-tuned changes could have important biological consequences [184]. Collectively, these findings suggest that the ER area constitutes part of the estrogenic rapid nongenomic pathway and that their splice variants, which are genomic changes, could undoubtedly affect the cooperation of the genomic and rapid nongenomic actions of estrogens.

In summary, although many questions remain to be resolved, there is substantial evidence that the biological effects of estrogenic actions constitute a complex interplay of genomic and nongenomic mechanisms and depend on the physiological and genetic context of the target cells. However, whether they rely on a cooperative or sequential modulatory system remains to be clarified. The combination of genomic and nongenomic mechanisms endows estrogens with considerable diversity, range, tissue, and power in modulating neural functions. Such cooperation may produce long-term changes in neuroplasticity. In this review, we highlighted the cellular and molecular mechanisms, including epigenetic modifications, synaptic protein synthesis, PTMs, and ER mutations to illustrate how estrogens induce changes in synaptic morphology and beneficial neuroprotection. Collectively, these literature investigations argue that the genomic and nongenomic estrogenic actions should probably be viewed as a unified mode.
